# Reply to Venkataraman, J.; Mokbel, K. Reconsidering the Interpretation of “Recurrence-Free Survival” After Mastectomy for DCIS. Comment on “Sae-sim et al. Tamoxifen Reduces Breast Cancer Recurrence in Women with DCIS Who Underwent Mastectomy. *Curr. Oncol*. 2026, *33*, 89”

**DOI:** 10.3390/curroncol33050248

**Published:** 2026-04-27

**Authors:** Netchanok Sae-sim, Norasate Samarnthai, Warapan Numprasit

**Affiliations:** 1 Division of Head Neck and Breast Surgery, Department of Surgery, Faculty of Medicine Siriraj Hospital, Mahidol University, Bangkok 10700, Thailand; netchanokpum@gmail.com; 2 Department of Pathology, Faculty of Medicine Siriraj Hospital, Mahidol University, Bangkok 10700, Thailand; norasatesmthai@yahoo.com

We thank Venkataraman and Mokbel [1] for their thoughtful and constructive comments regarding our article published in *Current Oncology* [2]. We appreciate the opportunity to clarify several aspects of our analysis and interpretation.

**Comment 1: Discrepancy in the Reported 10-Year Recurrence-Free Survival in the Abstract**

We thank the authors for identifying this inconsistency. The value reported in the Abstract (68.7%) was a typographical error. The correct 10-year recurrence-free survival for the non-endocrine therapy group is 77.9%, which is consistent with the Results section and the Kaplan–Meier figure. This error does not affect the statistical analyses or the reported hazard ratio. The Abstract will be corrected accordingly.

**Comment 2: Interpretation of Contralateral Events and Definition of Recurrence**

We agree that contralateral breast cancers represent new primary tumors rather than recurrence of the index DCIS after mastectomy. In our study, recurrence-free survival was defined as the time to any subsequent breast cancer event, including ipsilateral chest wall recurrence and contralateral breast cancer.

The inclusion of contralateral events reflects the broader concept of subsequent breast cancer risk, which is frequently used in studies evaluating endocrine therapy in DCIS populations. We acknowledge that the term “recurrence-free survival” may therefore be interpreted broadly and appreciate the suggestion that “subsequent breast cancer–free survival (SBCFS)” may be a more precise descriptor. We will clarify this endpoint definition in the revised manuscript.

Importantly, our findings should be interpreted within the context of tamoxifen’s established chemopreventive effect on contralateral breast cancer, which may contribute to the observed difference between groups.

**Comment 3: Small Number of Events**

We acknowledge that the total number of events in our cohort was limited (n = 16), which reflects the low baseline recurrence risk following mastectomy for DCIS. As a result, the hazard ratio is associated with a relatively wide confidence interval. This limitation was acknowledged in the Discussion section of the original manuscript. Our findings should therefore be interpreted as hypothesis-generating rather than definitive evidence of causality.

**Comment 4: Potential Confounding and Model Adjustment**

As noted by the commentators, our study was observational and treatment allocation was not randomized. Although we performed multivariable Cox regression to adjust for known clinicopathologic factors, residual confounding cannot be excluded.

Comedonecrosis was not included in the final multivariable model because it was not statistically significant, as shown in Table 2. In addition, given the limited number of events, inclusion of additional variables was restricted to avoid model overfitting and preserve model stability.

**Comment 5: Differences in Follow-Up Duration and Statistical Considerations**

We acknowledge that follow-up duration differed between groups. Kaplan–Meier analysis was used to account for variable follow-up time, which is standard practice in time-to-event analyses. However, we agree that differences in follow-up represent a potential source of bias and will further emphasize this limitation in the Discussion.

Regarding competing risks, we agree that alternative statistical approaches such as competing-risk regression may provide complementary insights. However, given the small number of events observed in our cohort, the stability of such models may also be limited.

## Conclusions

In summary, we appreciate the authors’ comments, which highlight important methodological considerations. We will correct the typographical error in the Abstract and clarify the endpoint definition and study limitations. Our findings should be interpreted as observational evidence suggesting a potential association between endocrine therapy and reduced subsequent breast cancer events following mastectomy for DCIS. Further studies with larger cohorts and prospective designs are warranted to confirm these findings.
